# A dirigent of the ring for strigolactone stereochemistry

**DOI:** 10.1073/pnas.2410953121

**Published:** 2024-08-12

**Authors:** Salim Al-Babili

**Affiliations:** ^a^The BioActives Lab, Biological and Environmental Science and Engineering Division, King Abdullah University of Science and Technology, Thuwal 23955-6900, Saudi Arabia; ^b^The Plant Science Program, Biological and Environmental Science and Engineering Division, King Abdullah University of Science and Technology, Thuwal 23955-6900, Saudi Arabia

Stereochemistry is crucial for the biosynthesis and functions of strigolactones (SLs), which act as plant hormones and rhizospheric signaling molecules (1). In this issue, Homma et al. (2) identified a dirigent domain-containing (DIR) protein as essential for governing the SL stereochemistry and established a model for the formation of the BC-ring in canonical SLs (see below).

SLs were first identified as compounds released by plant roots capable of inducing seed germination in root parasitic plants of the genus Striga (Latin for “witch”), commonly known as witchweeds (3), which pose a major agricultural problem around the Mediterranean Sea and in sub-Saharan Africa (4). Later on, it was shown that plants release SLs to communicate with soil-borne arbuscular mycorrhizal fungi (AMF) in order to establish a symbiosis with them (5). The arbuscular mycorrhizal symbiosis provides plants with minerals and water and rewards the fungal partner with reduced carbon (6). Around the same time, researchers interested in plant development were searching for a postulated hormone that inhibits shoot branching. Measurements of SL content in mutants affected in the biosynthesis of the presumed branching inhibitor, along with rescue experiments using the synthetic SL analog GR24, revealed that SLs are the missing inhibitory hormone (7, 8). Since this discovery, many hormonal functions of SLs have been identified, including the regulation of root architecture and senescence (1).

**Fig. 1.**
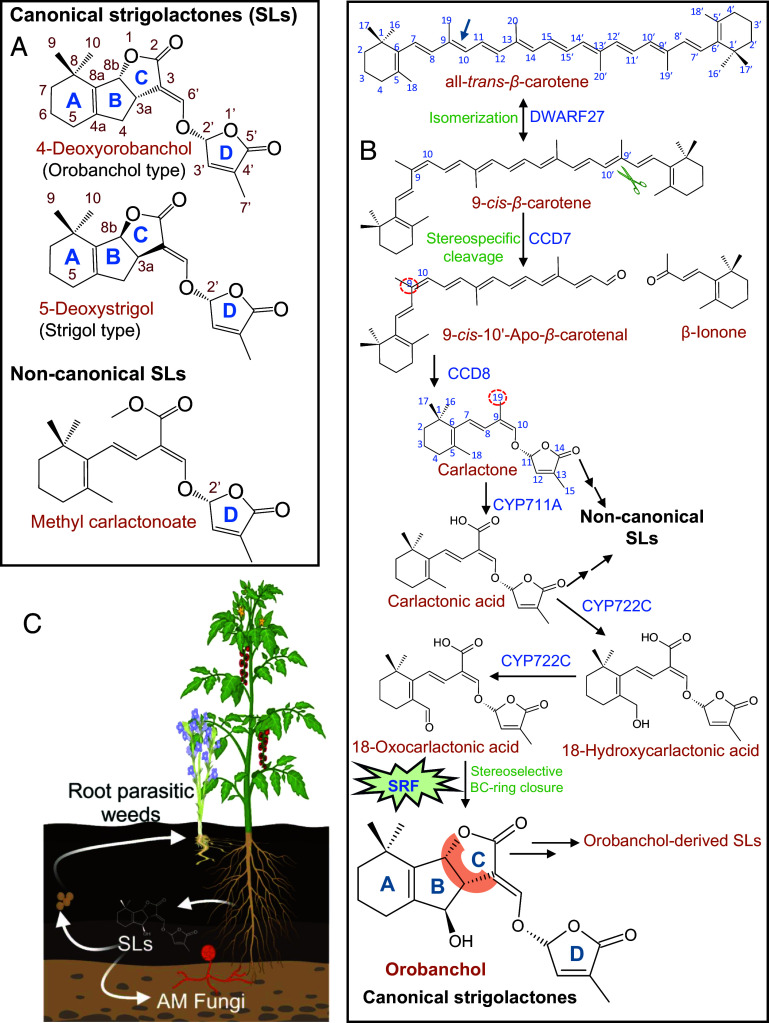
Biosynthesis and biological functions of strigolactones (SLs) in tomato (*Solanum lycopersicum*). (*A*) Structure of canonical (containing the characteristic tricyclic ABC-lactone) and noncanonical SLs. Canonical SLs are classified into orobanchol-type (α-oriented C-ring) and strigol-type (β-oriented C-ring). (*B*) SL biosynthesis in tomato. The enzyme DWARF27 isomerizes all-*trans*-β-carotene to 9-*cis*-β-carotene that is stereospecifically cleaved by CAROTENOID CLEAVAGE DIOXYGENASE (CCD7) to produce 9-*cis*-10′-Apo-β-carotenal. Further, CCD8 catalyzes the conversion of 9-*cis*-10′-Apo-β-carotenal to carlactone (numbering of atoms according to their position in β-carotene), which is oxidized by CYP711A to carlactonic acid (CLA) that gives rise to canonical and noncanonical SLs. CYP722C converts CLA into 18-hydroxycarlactonic acid and 18-oxocarlactonic acid. The stereo-selective BC-ring-forming factor (SRF; SlDIR) directs the C-ring formation toward orobanchol, which would otherwise also lead to the strigol-type SL *ent*-2′-*epi*-orobanchol. Orobanchol is the precursor of a series of SLs in tomato. (*C*) Orobanchol and other released SLs are important for rhizospheric communication with AMF and are abused as germination stimulants for root parasitic weeds such as Phelipanche species of the Orobanchaceae family. Panel *C* was created partially with the help of BioRender.com.

Structurally, SLs are defined as carotenoid derivatives containing a butenolide ring (D-ring) coupled to a second moiety through an enol ether bridge in the *R*-configuration (Fig. 1) (3). As of now, approximately 35 natural SLs have been characterized (1). Depending on the second moiety, SLs are divided into canonical SLs that harbor a tricyclic lactone (called the ABC-ring) and noncanonical SLs that contain variable structures (9). Stereochemistry further defines two types of canonical SLs (Fig. 1): the strigol-type SLs, with the C-ring in the 8b*S*-configuration (up/β-oriented), and the orobanchol-type SLs, with the C-ring in the 8b*R*-configuration (down/α-oriented). Besides the common noncanonical SLs, some plant species, such as tomato and cotton, produce either orobanchol-type or strigol-type SLs, respectively (2).

The diversity of SL functions and structures raises questions about whether specific SLs have distinct biological roles. Studies with rice and tomato mutants indicate that canonical SLs primarily function in rhizospheric communication rather than in the regulation of shoot architecture (10–12). Additionally, rhizospheric partners may prefer certain SL types. In sorghum and maize, altering the pattern of released SLs can reduce Striga seed germination and infection (13, 14). For AMF, orobanchol shows the highest activity in inducing hyphal branching and may be a more effective attractant for symbiosis establishment (15).

SLs arise from all-*trans*-β-carotene via carlactone, which is modified by cytochrome P450 (CYP450) and other enzymes into various SLs [(1); Fig. 1]. The conversion of carlactone into carlactonoic acid (CLA) by CYP711A is a common step in the formation of both canonical and noncanonical SLs (1). The reactions catalyzed by the Carotenoid Cleavage Dioxygenase 8 (CCD8; Fig. 1) ensure that carlactone, and thus, all SLs, possess the correct stereo-configuration at the enol ether bridge (3). But what about the sereo-configuration at the C-ring?

In rice, which produces orobanchol-type SLs, the CYP711 OsMAX900 catalyzes the stereospecific BC-ring closure, resulting in 4-deoxyorobanchol that can be converted by the homolog OsMAX1400 into orobanchol (16). It is proposed that CYP7911A2 generates 18-hydroxy-carlactonoic acid (18-OH-CLA) as an intermediate and stereospecifically converts it into 4-deoxyorobanchol. In the absence of an enzyme controlling the stereochemistry, 18-OH-CLA could spontaneously form the BC-ring, yielding both 5-deoxystrigol and 4-deoxyorobanchol (16). Similarly, in cotton that produces strigol-type SLs, CYP722C enzymes mediate the stereospecific conversion of CLA into 5-deoxystrigol.

Unlike rice, tomato and cowpea synthesize orobanchol directly from CLA, bypassing the precursor 4-deoxyorobanchol, using CYP722C enzymes (11), which, however, cannot determine the stereochemistry of the BC-ring formation (2). Incubations of CLA with tomato SlCYP722C or cowpea VuCYP722C yielded orobanchol and its diastereomer, *ent*-2′-*epi*-orobanchol that has the opposite C-ring configuration. The recent study by Homma et al. carefully investigated the formation of the BC ring of orobanchol in tomato and identifies the factors that assist SlCYP722C in producing orobanchol in the correct stereo-configuration (2).

Homma et al. (2) initially identified 18-OH-CLA and 18-oxocarlactonoic acid (18-oxo-CLA) as the products from the conversion of CLA by SlCYP722. Expanding on their previous study of BC-ring formation, which proposed an acid-mediated, spontaneous, sequential cyclization of 18-oxo-CLA via a conrotatory 4π-electrocyclic reaction (ECR) (17), the researchers acidified the assay at its conclusion. They observed the formation of two diastereomers of orobanchol, supporting their model and suggesting that orobanchol BC-ring formation occurs through an intermediate with a delocalized positive charge resulting from proton uptake. Consequently, the authors proposed that the SlCYP722C catalysis is limited to the two-step oxidation of CLA to 18-oxo-CLA, which can spontaneously form orobanchol and its diastereomer. This model necessitates the involvement of another factor termed the “stereoselective BC-ring-forming factor (SRF),” which ensures the correct stereo-configuration of orobanchol.

Assuming SRF expression is coordinated with SL biosynthetic genes, which are upregulated by phosphate deficiency (18), the authors analyzed related tomato and cowpea transcriptome datasets and identified DIR proteins from tomato and cowpea as candidates for the SRF (2). DIR proteins act as templates that control the stereochemistry of reactions. In lignin biosynthesis, they facilitate the stereo-selective coupling of phenoxy radicals (19). The authors incubated recombinant tomato SlDIR (Solyc01g059900) with 18-oxo-CLA and observed the formation of orobanchol, but not its diastereomer. This experiment demonstrated that the identified SlDIR is an SRF, mediating the stereospecific closure of the BC-ring towards orobanchol. Corresponding loss-of-function tomato mutants generated using CRISPR-Cas9 showed equal amounts of orobanchol and *ent*-2′-*epi*-orobanchol, suggesting that SlDIR plays a crucial role as an SRF in planta. Interestingly, the mutants showed also a loss of SLs derived from orobanchol (Fig. 1), indicating that the synthesis of canonical SLs in tomato may require a structural arrangement as a metabolon involving SlSRF.

The study of Homma et al. provides valuable insights into the enzymatic processes involved in SL biosynthesis and opens up new possibilities for agricultural applications aimed at improving plant interactions with their environment and managing parasitic weed problems.

The authors employed quantum mechanics calculations to delve deeper into SlSRF’s catalysis, revealing that protonation of 18-oxo-CLA and specific conformational selection lead to orobanchol formation (2). Using AlphaFold2, they predicted SlSRF’s tertiary structure and elucidated its catalytic mechanism. This involved manual docking of 18-oxo-CLA, molecular dynamics simulations for complex stability, and density functional theory calculations integrating molecular orbital and mechanics. Their integrated approach pinpointed critical residues governing protonation and substrate coordination, ensuring strict control over 18-oxo-CLA’s conformation to exclusively yield orobanchol. Seven mutant variants targeting these residues showed varying degrees of activity loss, with D37A exhibiting complete inactivity, affirming its role as the crucial proton donor initiating the reaction.

In summary, Homma et al. utilized a combination of experimental and computational approaches to address how dicot plants synthesize orobanchol with the correct stereochemistry from CLA. Their work established a catalytic mechanism of BC-ring formation and identified a DIR as the SRF. This SRF facilitates the ring formation, ensuring the correct stereo-configuration of the product in a reaction that would otherwise produce two diastereomers. The recruitment of an SRF to avoid producing a mixture of orobanchol diastereomers suggests that releasing orobanchol as the sole diastereomer, along with its derivatives, may enhance communication with and recruitment of beneficial rhizospheric partners. The identification of the SRF provides an additional tool for rhizosphere engineering, which could be instrumental in reducing infestation by root parasitic plants that depend on root-released SLs as germination signals.

Overall, the study of Homma et al. provides valuable insights into the enzymatic processes involved in SL biosynthesis and opens up new possibilities for agricultural applications aimed at improving plant interactions with their environment and managing parasitic weed problems.
